# *In vivo* Bioluminescence Imaging Used to Monitor Disease Activity and Therapeutic Response in a Mouse Model of Tauopathy

**DOI:** 10.3389/fnagi.2019.00252

**Published:** 2019-09-12

**Authors:** Ambrose A. Dunn-Meynell, Peter Dowling, Michelle Marchese, Esther Rodriguez, Benjamin Blumberg, Yun-Beom Choi, Deeya Gaindh, Wei Lu

**Affiliations:** ^1^Neurology Service, VA New Jersey Health Care System, East Orange, NJ, United States; ^2^Department of Neurology and Neurosciences, Rutgers New Jersey Medical School, The State University of New Jersey, Newark, NJ, United States; ^3^Department of Neurology, Rutgers New Jersey Medical School, The State University of New Jersey, Newark, NJ, United States

**Keywords:** gliosis, glial fibrillary acidic protein, transgenic, PS19, erythropoietin, P301S, JM4, Alzheimer’s disease

## Abstract

Many studies of tauopathy use transgenic mice that overexpress the P301S mutant form of tau. Neuronal damage in these mice is associated with astrogliosis and induction of glial fibrillary acidic protein (GFAP) expression. GFAP-luc transgenic mice express firefly luciferase under the GFAP promoter, allowing bioluminescence to be measured non-invasively as a surrogate biomarker for astrogliosis. We bred double transgenic mice possessing both P301S and GFAP-luc cassettes and compared them to control mice bearing only the GFAP-luc transgene. We used serial bioluminescent images to define the onset and the time course of astrogliosis in these mice and this was correlated with the development of clinical deficit. Mice containing both GFAP-luc and P301S transgenes showed increased luminescence indicative of astroglial activation in the brain and spinal cord. Starting at 5 months old, the onset of clinical deterioration in these mice corresponded closely to the initial rise in the luminescent signal. Post mortem analysis showed the elevated luminescence was correlated with hyperphosphorylated tau deposition in the hippocampus of double transgenic mice. We used this method to determine the therapeutic effect of JM4 peptide [a small peptide immunomodulatory agent derived from human erythropoietin (EPO)] on double transgenic mice. JM4 treatment significantly decreased the intensity of luminescence, neurological deficit and hyperphosphorylated tau in mice with both the P301S and GFAP-luc transgenes. These findings indicate that bioluminescence imaging (BLI) is a powerful tool for quantifying GFAP expression in living P301S mice and can be used as a noninvasive biomarker of tau-induced neurodegeneration in preclinical therapeutic trials.

## Introduction

Tauopathies including Alzheimer’s disease (AD), frontotemporal dementia (FTD), progressive supranuclear palsy and corticobasal degeneration are marked by abnormal hyperphosphorylation of the microtubule-associated protein tau. As well as their devastating neurological symptoms, neuropathological changes occur in tauopathies including chronic neuroinflammation with microglial and astrocytic gliosis.

Substitutions within the gene encoding tau may trigger tauopathies in humans. A single point mutation at the P301S site causes FTD with parkinsonism linked to chromosome 17 (FTDP-17; Bugiani et al., 1999; Baba et al., 2007). Transgenic mice overexpressing P301S mutant human tau have been widely used as a model of AD and other tauopathies. These mice display progressive motor deficits (Hurtado et al., 2010; Scattoni et al., 2010), shortened life span (Yoshiyama et al., 2007), and increased levels of hyperphosphorylated tau in the cortex, hippocampus, cerebellum, and spinal cord (Allen et al., 2002; Yoshiyama et al., 2007; Hurtado et al., 2010; Chalermpalanupap et al., 2018).

Several studies have also shown rises in glial fibrillary acidic protein (GFAP) expression in these mice, indicative of astroglial activation (Yoshiyama et al., 2007; Hampton et al., 2010; López-González et al., 2015). However, these studies were limited in their ability to follow the time course of gliosis due to the need to sacrifice animals at each time point examined. The primary objective of the current study is to use *in vivo* bioluminescence imaging (BLI) techniques in P301S mice carrying the GFAP-luc transgene to quantify gliosis at numerous time points in order to allow for the study of astrocytic gliosis during disease progression.

BLI is a sensitive non-invasive imaging technique that allows quantifiable examination of gene expression in living animals. It is known for its broad clinical applicability in many areas of research (Sadikot and Blackwell, 2005; Dothager et al., 2009). In GFAP-luc mice, the firefly luciferase gene is driven off the GFAP promotor allowing bioluminescence to be used as a measure of gliosis. This model has previously been used to longitudinally quantify GFAP expression following central nervous system (CNS) inflammation (Cordeau et al., 2008; Cordeau and Kriz, 2012; Sydow et al., 2016). BLI signals in GFAP-luc mice have also been shown to correlate with amyloid-beta deposition in the brain, and BLI may serve as a possible tool in the study of amyloid beta directed therapeutics (Watts et al., 2011).

Our secondary objective was to use the GFAP-luc BLI model to investigate the effectiveness of a new erythropoietin (EPO) based therapeutic agent in the treatment of tauopathies. EPO is a pleiotropic cytokine shown to have neuroprotective and anti-inflammatory effects in a variety of animal models; however, complications associated with its hematopoietic properties limit its use in clinical practice (Dicato, 2008). Our group previously generated a small EPO derived peptide (JM4) and has recently obtained FDA approval for its use as an Investigational New Drug (IND#141060). We have found that JM4 is highly neuroprotective while avoiding the side effects of the whole molecule (Yuan et al., 2015). JM4 has been shown to cross the blood-brain barrier (Wang et al., 2016), downregulate neuroinflammation in experimental autoimmune encephalomyelitis (Yuan et al., 2015), and has shown remarkably strong beneficial effects in acute traumatic brain injury (Wang et al., 2016). Chronic neuroinflammation is one of the characteristic hallmarks of AD (Heneka et al., 2015; Shadfar et al., 2015; Van Eldick et al., 2016). We hypothesized that JM4 would have therapeutic effects in the P301S mouse model of AD and tauopathies, and that these effects could be tracked *in vivo* using BLI.

## Materials and Methods

### Animals

Female mice heterozygous for the human P301S tau transgene (B6;C3-Tg(Prnp-MAPT*P301S)PS19Vle/Jackson Laboratories, Wilmington, MA, USA) were crossed with male mice heterozygous for the GFAP-luc transgene (FVB/N-Tg [GFAP-luc+/−], Xenogen Corp., Alameda, CA, USA). The resulting F1 offspring were genotyped using tail snip polymerase chain reaction (PCR) to identify transgenic male and female mice with both the bioluminescent GFAP-luc and the P301S transgenes (P301S mice).

Control mice were littermates heterozygous for the GFAP-luc transgene alone and not possessing the P301S tau transgene. In addition, five wild type B6C3 mice were used for post mortem examination of AT8 immunohistochemistry (IHC; WT control). Animal use was approved and monitored by the Institutional Animal Care and Use Committee of the Veterans Administration New Jersey Health Care System (VANJHCS), East Orange Campus. This group ensured that the research complied with the ethical guidelines of the United States Public Health Service and the Office of Laboratory Animal Welfare. All animal work was performed within the VANJHCS, East Orange, which is fully accredited by the Association for Assessment and Accreditation of Laboratory Animal Care.

### JM4 Peptide

JM4 is a small EPO-derived peptide (N28-GCAEHCSLNENITVPDTKV-46C), synthesized and purified by United Biochemical Research (Seattle, WA, USA) and cyclized for stability as previously described (Yuan et al., 2015). JM4 was dissolved in phosphate buffered saline (PBS) at 2 mg/ml and aliquots were stored at −20°C for up to 2 months.

### Experimental Design

Experiments were designed to appraise the gliosis occurring in P301S mice using bioluminescence. We further compared the development of gliosis to the onset of neurological deficits that characterize the model to determine if BLI could be used as a predictor of functional deficits in the disease. At sacrifice, we compared immunocytochemically defined GFAP levels in spinal cord with bioluminescence levels to verify the validity of the BLI technique. The study also examined the effect of our novel EPO derived peptide, JM4 on disease progression. We examined each of these variables in JM4 treated P301S mice to determine if the drug could block disease progression. Further, since tauopathy is a key characteristic of neurodegeneration in P301S mouse we appraised hyperphosphorylated tau immunocytochemically to examine whether JM4 could block its buildup and correlated tau levels with BLI.

**Figure 1:**
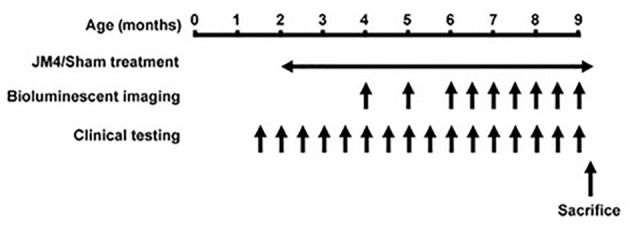
Timeline of the experiments. Animals received JM4 or sham treatment from 2 months of age until sacrificed. Bioluminescence imaging (BLI) was performed 1–2 times per month between 4 and 9 months old. Neurological testing was performed twice per month from 1.5 month old. If a mouse reached its humane endpoint during the treatment period it was sacrificed along with its matched Control. All other mice were sacrificed after the final BLI measurement.

Three treatment groups carrying the bioluminescent GFAP-luc transgene were examined: P301S mice treated with JM4 peptide (P301S JM4); P301S mice treated with PBS vehicle (P301S SHAM); and untreated animals that lacked the P301S gene (Control). Treatment was initiated at 2 months of age and continued until the end of the experiment (Figure 1). JM4 treated mice received 10 μg of JM4 in 100 μl PBS subcutaneously, 5 days per week. This dose was found to be optimal in studies of the efficacy of JM4 to treat traumatic brain injury (Wang et al., 2016). Sham mice received PBS injections without JM4.

Pairs of P301S mice (matched by gender where possible) were drawn from individual litters, and one of each pair was assigned to the JM4 treatment or sham treatment groups for a total of eight pairs (Table 1). If one mouse from a given pair reached its humane endpoint due to disease progression, then both mice in the pair were sacrificed in order to allow comparison of AT8 IHC in animals of equivalent age. IHC was also examined in wild type mice of the same gender and age. Untreated GFAP-luc mice were used as Controls for BLI.

**Table 1:** Number of male (M) and female (F) animals used in experiments.

Transgenic type and treatment	Treatment	Bioluminescence imaging/clinical score	Tau immuno-histochemistry	GFAP immuno-histochemistry
P301S	JM4	8 (6 F, 2 M)	5 (3 F, 2 M)	4 (3 M, 1 F)
P301S	Sham	8 (6 F 2 M)	5 (3 F, 2 M)	2 (1 M, 1 F)
Control	None	5 (3 F, 2 M)	0	
Wild type control	None	0	5 (3 F, 2 M)	

The numbers of subjects examined was based on power analysis performed using GPower 3.1.9.2. using effect sizes derived from prior experience with P301S mice and JM4 treatment. Numbers were optimized to provide statistical proof that peak BLI levels differed between P301S mice and controls, and that JM4 treated P301S animals differed from untreated P301S mice. We anticipated a smaller effect size when comparing the two P301S groups, therefore we used the largest number of animals in each these groups (*N* = 8 each). Conversely, we expected a stronger effect size when comparing controls to untreated P301S mice, therefore an *N* of 5 was used in the control group.

### Bioluminescence Imaging

BLI of GFAP-luc was initiated at 4 months of age and continued once or twice per month to 9 months of age (Figure 1). The imaging procedure was performed as described by Luo et al. (2008) with minor modifications. Briefly, luminescence was quantified using the *in vivo* Imaging System (IVIS, Lumina, Caliper Life Science, Waltham, MA, USA), consisting of a cold charge-coupled device camera mounted in a dark box. The mice were injected intraperitoneally with 3 mg/kg D-luciferin 10–15 min prior to imaging. They were then anesthetized with isoflurane (1.5%–2.0% in oxygen) for the remainder of the process (5–6 min) and mounted with a standard orientation in the imager. Luminescence was measured in units of photons/s/cm^2^/steradian using Living Image Version 3.1 software (Caliper Life sciences, Waltham, MA, USA) and integrated over 2 min. For quantification, photons were measured over a “region of interest” (ROI) that covered 1.3 cm^2^ over the brain and 7.8 cm^2^ over the spinal cord. We expected that there might be variability in BLI intensity between sessions associated with the speed of uptake and metabolism of D-luciferin after IP injection. We, therefore, made a reference measurement over a 0.7 cm^2^ area above the left ear on each imaging day. We then normalized the data in the ROI by presenting it as a ratio of BLI in the left ear × 100 in each imaging session. We were unable to use repeated measures analysis of variance (ANOVA) or a mixed model to produce a single global statistic comparing overall bioluminescent data due to loss of a subject and its matched pair during the experimental course. Instead, we performed a global statistical analysis based upon a single measure (the highest ROI/ear ratio observed) for brain and spinal cord in each subject. ANOVAs with *post hoc* tests were performed to compare bioluminescence at individual time points.

### Neurological Assessment

A modified version of the limb clasping test described by Miller et al. (2008) was used to quantify the limb retraction and clasping found in this transgenic mouse model (Yoshiyama et al., 2007). This was performed on JM4 and sham-treated P301S mice beginning at 1.5 months of age and continued to 9 months of age (Figure 1). Mice were suspended by their tails for 30 s and clinical scores were assigned on the following scale: 0 = no functional deficit; 1 = hind paw clasping for at least 10 s; 2 = retraction of one or both hind limbs for the full 30 s; 3 = failure to lift the hind limbs from the body due to weakness; 4 = total hind limb paralysis; 5 = paralysis of all 4 limbs; 6 = moribund. Deficit was appraised by two observers, both of whom were unaware of the animal’s experimental group and treatment. Each of the observers assigned scores based on their own assessment and was unaware of the score assigned by the other. The age when mice developed marked neurological deficits (i.e., reached or exceeded a clinical score of 2) was used as a single measure of clinical deterioration for statistical analysis.

### Humane Endpoints

Since the P301S transgenic model is known to have a shortened life expectancy (Yoshiyama et al., 2007) we set an experimental endpoint before significant mortality was anticipated (9–10 months of age). In case any animals showed early disease progression, a humane endpoint was established and approved by the institution’s animal care and use committee (>30% weight loss, persistent pain or distress severe neurologic disorders compromising the subjects ability to reach food and water, or intractable skin or urinary infections). Animals were monitored daily, and one subject was euthanized at its humane endpoint during the course of the study.

### Immunohistochemistry Studies

After the final BLI measurement, mice were deeply anesthetized with ketamine (120 mg/kg) and Xylazine (15 mg/kg) and sacrificed by transcardiac perfusion with ice-cold saline followed by ice-cold 4% paraformaldehyde in PBS. Brains and spinal cords were harvested and post-fixed in 4% paraformaldehyde at 4°C for 2 h.

All IHC procedures were performed at room temperature in PBS based solutions (pH 7.4) except where otherwise specified.

Tau IHC was studied in five randomly selected pairs of JM4 and Sham treated P301S mice as well as five wild type B6C3 mice. Brains were examined with monoclonal mouse anti-human Phospho PHF-tau pSER202+Thr205 monoclonal antibody (AT8, Thermo Scientific MN1020, Antibody Registry ID AB_223647). This antibody was selected as it demonstrates high specificity for Phospho-tau in mouse models of AD (Petry et al., 2014). Brains were immersed in 20% sucrose in PBS at 4°C overnight and then frozen. Eight micrometer thick coronal sections were collected and post-fixed in acetone at −20°C for 10 min then air dried and stored at −80°C. Slides were washed for 5 min then blocked in 0.3% Hydrogen peroxide for 10 min. They were then washed three times (10 min each), blocked in 2% Normal horse serum for 10 mins and then incubated overnight at 4°C in 1:20 AT8 antibody. The next day slides were incubated in 1:250 biotinylated anti-mouse igG (Vectastain Peroxidase Mouse kit PK-4002), washed three times for 10 min and then reacted with Vectastain peroxidase ABC reagent for 30 min. After three more 10 min washes, slides were reacted with tyramide signal amplification Cy3 reagent (Perkin Elmer Life sciences NEL704A) diluted 1:100 in the buffer provided by the kit manufacturer. Finally slides were washed three times in distilled water and cover-slipped in glycerol-PBS.

GFAP IHC was also examined in a subset of P301S mice using DAKO polyclonal rabbit anti GFAP antibody (Dako Z0334, Antibody Registry ID AB_10013382). This antibody has been widely used to demonstrate GFAP (for example Costa et al., 2019; Fuentes-Santamaria et al., 2019). Spinal cords from six P301S mice (two sham treated and four JM4 treated) were paraffin embedded and sectioned at 7 μm. After deparaffinizing and washing three times for 10 min, antigens were unmasked using Target Unmasking Fluid (Pan Path) in distilled water at 95°C for 10 min. After three further 10 min washes slides were blocked with 2% Normal goat serum before overnight incubation at 4°C in 1:1,000 anti-GFAP. The next day, slides were washed three times for 10 min then visualized in 1:200 Cy3 conjugated anti-rabbit igG (Jackson 711-165-152) and rewashed three times for 10 min before cover slipping in glycerol-PBS. Control sections without primary antibody were also examined.

Hippocampal tauopathy is a hallmark of the P301S mouse at 9 months old when animals were sacrificed (Yoshiyama et al., 2007). Accordingly, IHC staining was examined quantitatively in this structure. AT8 was measured in three non-adjacent sections through the anterior portion of the hippocampus. A 792 × 492 μm area comprising the majority of the hippocampus on one side of the brain was measured in each section. The percentage of AT8 labeled structures in this area was measured using Image-Pro Premier (Media Cybernetics, Rockville, MD, USA). Label was defined as the percent total area where fluorescence exceeded an arbitrarily set intensity limit that was held constant in all subjects. GFAP immunoreactivity was examined using similar techniques in a total of 15 sections per subject spanning the cervical, thoracic and lumbar spinal cord with a fixed area of 515 × 467 μm in each.

### Statistical Analysis

**Figure 2:**
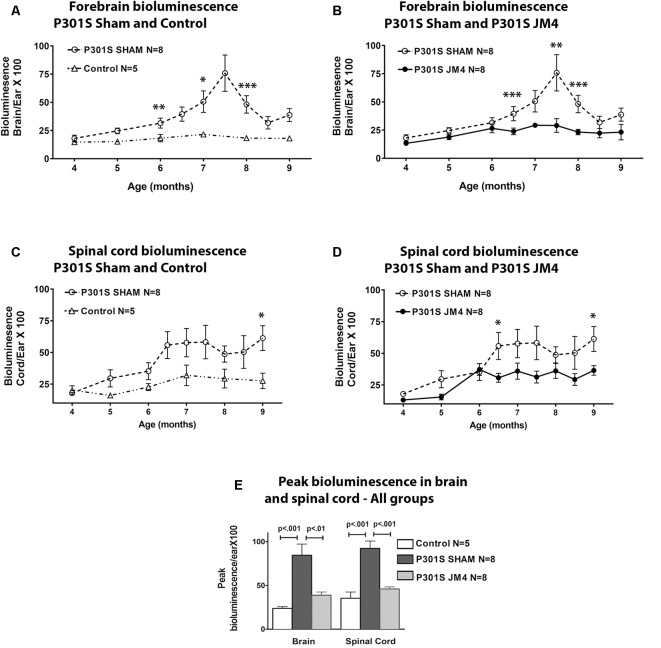
Glial fibrillary acidic protein (GFAP) bioluminescence in Control and P301S mice. BLI (mean ± SE) in Control (*n* = 5), Sham treated P301S (P301S SHAM *n* = 8) and JM4 treated P301S mice (P301S JM4 *n* = 8). Panels **(A–D)** show BLI levels over time in brain and spinal cord. Asterisks show time points where BLI levels differ significantly between the two groups plotted in each graph (*p < 0.05, **p < 0.025, ***p < 0.01). There was no significant difference between P301S JM4 and Control animals. **(A)** P301S SHAM mice show a marked increase in forebrain signals after 4 months of age vs. Control mice that exhibit low signal for the entire time course. **(B)** The same P301S SHAM mice show markedly elevated forebrain BLI vs. JM4 treated P301S mice. **(C)** P301S Sham animals show a large increase in spinal cord BLI at 6.5 months of age compared to Control animals. **(D)** The same P301S SHAM mice show elevated spinal cord BLI relative to JM4 treated P301S mice starting at 6.5 months. **(E)** Peak bioluminescence was significantly higher in P301S SHAM mice than Control mice in both the brain (*p* < 0.001) and spinal cord (*p* < 0.001). Peak bioluminescence in P301S JM4 animals was similar to Control mice and significantly lower than P301S SHAM in both brain (*p* < 0.01) and spinal cord (*p* < 0.001).

The analysis was performed using GraphPad Prism 6.0 or 7.0 software. Normal distribution of data was verified using the Kolmogorov–Smirnov normality test. When comparing three groups of subjects (Figure 2; peak BLI levels and most of the individual time points and Figure 7; AT8 staining), we used a one-way ANOVA. After demonstrating overall significance, *post hoc* Tukey’s tests with Bonferroni correction were used for inter-group comparisons. Where only two groups were compared, an unpaired *t*-test was used. Linear regression was used to correlate the intensity of bioluminescence with levels of GFAP and AT8 IHC. The datasets generated for this study are presented in the Supplementary Material accompanying this article.

**Figure 3:**
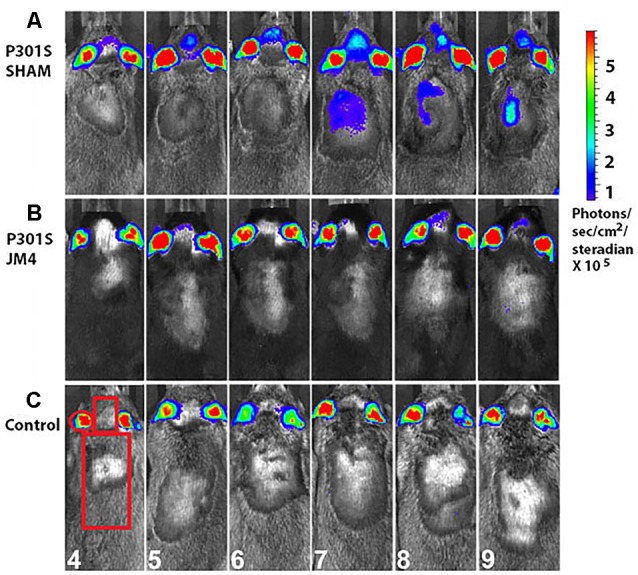
Monthly bioluminescence signals in the three treatment groups. Regions of interest (ROI) over the ear, brain and spinal cord were used for quantification of luminescence signals. **(A)** A sham-treated P301S mouse shows GFAP signals in the forebrain at 4 months followed by the onset of spinal cord signal 3 months later. **(B)** A JM4-treated P301S littermate shows only slight luminescence in the forebrain. **(C)** A Control mouse shows minimal luminescence signal in the brain and spinal cord throughout the experiment. Regions of interest and ages of the animals in months are shown in panel **(C)**.

**Figure 4:**
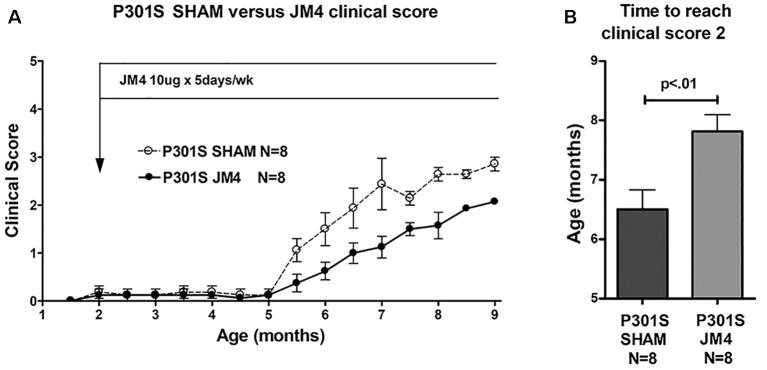
Clinical scores of JM4 and sham-treated P301S mice. **(A)** Both groups showed no or little clinical deficit until 5 months. Thereafter P301S SHAM mice deteriorated faster than P301S JM4 mice. **(B)** The time to reach a clinical score of 2 was significantly longer in JM4 treated mice compared to sham treated (*p* < 0.01).

**Figure 5:**
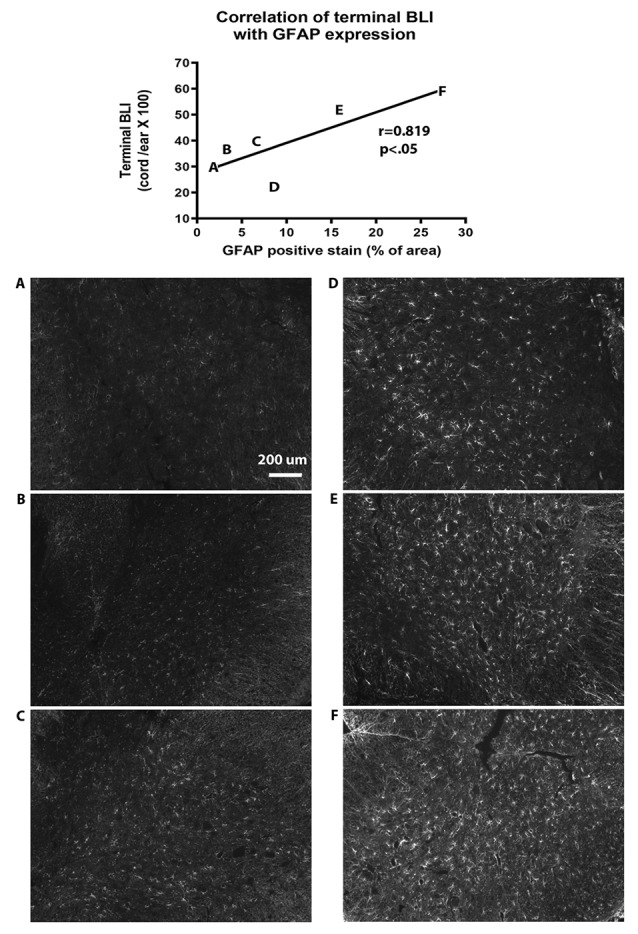
Correlation of bioluminescence with GFAP expression in the spinal cord. The diagram at the top of the figure shows that GFAP immunohistochemistry (IHC) is correlated with terminal BLI readings (*y* = 27.3 + 1.18 ×, *r* = 0.819, *p* < 0.05 by linear regression analysis). Letters on the graph represent results from individual animals. **(A–F)** Photomicrographs of GFAP IHC from each of the corresponding animals are shown below the graph.

**Figure 6:**
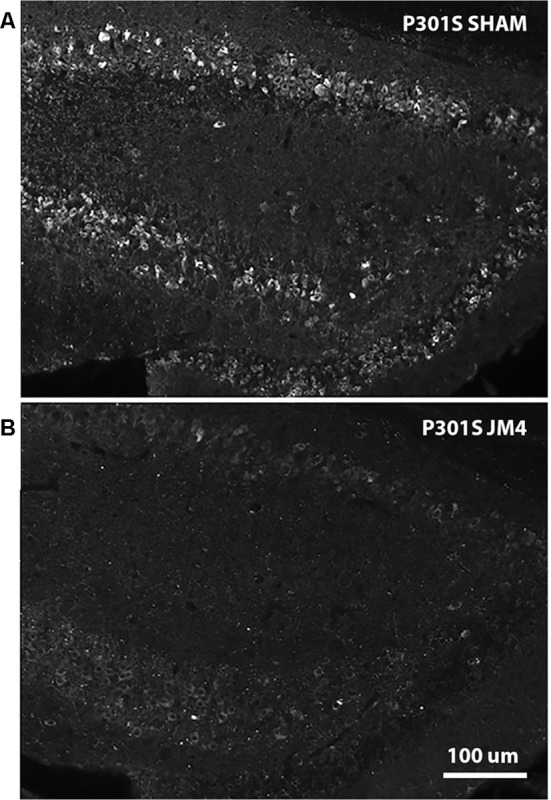
AT8 IHC in hippocampal sections of Sham vs. JM4 treated P301S mouse brains. **(A)** Sham treated mouse brain shows abundant strongly positive hyperphosphorylated tau aggregates in hippocampal nerve cells. **(B)** In marked contrast, the JM4 treated mouse shows dramatically reduced levels of tau immunostaining. Photographs were adjusted for optimal brightness and contrast using Adobe Photoshop. Every pixel in both panels was adjusted identically.

**Figure 7:**
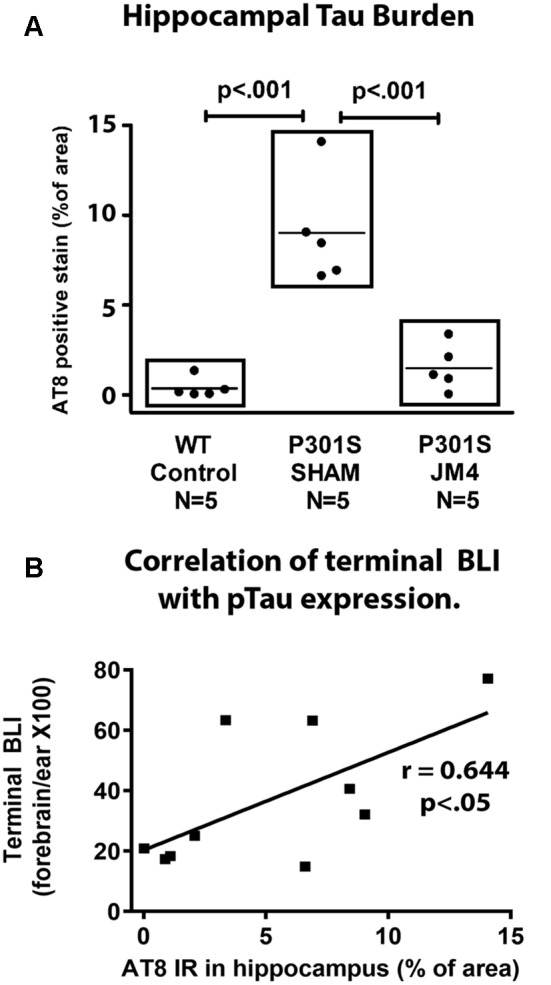
Quantitative measurements of AT8 staining and their correlation with Bioluminesence. **(A)** IHC was measured in the hippocampus of wild type controls (WT control), and Sham or JM4 treated P301S mice (*n* = 5 per group). Each point represents a single mouse. P301S SHAM mice had significantly more tau than WT controls (*p* < 0.001). P301S JM4 animals had greatly reduced tau burden relative to P301S SHAM mice (*p* < 0.001) and did not differ significantly from WT controls. **(B)** Results from the P301S SHAM and P301S JM4 animals are plotted against their terminal forebrain BLI. There is a significant correlation between the two measures (*y* = 20.32 + 3.235 ×, *r* = 0.644, *p* < 0.05).

## Results

We employed a simple non-invasive imaging technique to examine the onset and progression of astrocytosis in a transgenic mouse model. First, we compared the evolution of luminescent signals in the brain and spinal cord of sham-treated P301S vs. Control mice starting at the pre-symptomatic age of 4 months through 9 months old. Luminescence increased beyond the age of 4 months in P301S SHAM mice when compared to Control mice (Figures 2A,C, 3A,C). The Control wild-type animals, as expected, showed comparatively low luminescence throughout the time course of the study in both the brain and spinal cord. The sham-treated P301S animals showed increasing luminescence beyond 4 months of age both in the brain and spinal cord. After 7.5 months of age, brain luminescence decreased but remained above Control levels. In the spinal cord, the luminescence signal in the sham-treated P301S group increased at 5 months and remained constantly elevated relative to the Control animals. Statistical analysis demonstrated higher peak luminescence in P301S mice compared to Control in both the brain (*p* < 0.001) and spinal cord (*p* < 0.001; Figure 2E).

We next investigated the effect of JM4 therapy on luminescence in P301S mice (Figures 2B,D,E, 3B). The dramatic rise in brain luminescence observed in sham-treated P301S animals after 6 months of age was not observed in JM4 treated mice. Peak luminescence in the brain of JM4 treated P301S mice was significantly (*p* < 0.01) lower than sham-treated P301S animals, and did not differ significantly from untreated Control mice (Figure 2E). In the spinal cord, the rise in luminescence was not apparent in the JM4 treated group until 6 months of age (Figure 2D) and was subsequently markedly reduced compared to the sham-treated group. Peak luminescence in the spinal cord of JM4 treated P301S mice was significantly lower than sham-treated P301S animals (*p* < 0.001; Figure 2E). Conversely, JM4 treated P301S mice showed similar overall levels of spinal cord luminescence to untreated Control mice, and peak cord luminescence in the JM4 treated P301S animals did not differ significantly from untreated Control mice (Figure 2E).

To determine if a change in luciferase reporter activity correlated with neurological status, we serially scored clinical deficit in sham and JM4 treated P301S mice. Animals in both groups were clinically asymptomatic until 5 months of age. P301S sham-treated mice showed abrupt onset of neurologic symptoms at 5.5 months (Figure 4A) when they began to demonstrate hind limb clasping. Deficits progressed to hind limb retraction followed by paralysis at 7–9 months of age. JM4 treated P301S mice also showed deficits starting at 5.5 months, however, at each subsequent time point, deficits appeared to be lower than those seen in sham-treated mice. JM4 treated mice reached a clinical score of 2 significantly later than sham-treated P301S animals (Figure 4B, *p* < 0.01). Control mice showed no evidence of neurological deterioration by the time of sacrifice (clinical score zero).

In order to verify that BLI was representative of GFAP expression, we compared terminal BLI measurements with post-mortem immunohistochemical analysis of GFAP expression in the spinal cord. This confirmed that BLI levels were paralleled by levels of GFAP expression (Figure 5).

To confirm that luminescent changes were accompanied by progression of pathology, distribution of hyperphosphorylated tau in the brain was quantified post mortem by IHC using the phosphorylation-dependent anti-tau antibody AT8. Figure 6A illustrates abundant strongly positive hyperphosphorylated tau aggregates in hippocampal nerve cell bodies and their processes in a sham-treated P301S mouse. In contrast, there was a dramatic reduction in the abundance of tau in a JM4 treated mouse at a similar age (Figure 6B). Figure 7A shows the percent area within the hippocampus that displayed AT8 staining in JM4 and sham-treated P301S and untreated wild type B6C3 mice. As expected, WT control mice showed minimal AT8 staining. Global ANOVA with *post hoc* tests revealed that the area occupied by AT8 staining was significantly elevated in sham-treated P301S mice relative to both JM4 treated P301S (*p* < 0.001) and to WT control mice (*p* < 0.001). The two latter groups did not differ significantly from each other. Regression analysis showed that the intensity of terminal BLI in the forebrain was significantly correlated with the amount of AT8 staining (Figure 7B).

## Discussion

The principal strength of our study is our demonstration that BLI can be used as a noninvasive quantitative biomarker to serially monitor pathological changes in the brain of the P301S transgenic mouse model of AD. We also show that the increase in luminescence parallels the onset of clinical signs and utilized this technique to study the long-term therapeutic benefits of the small EPO-derived peptide, JM4.

We used IHC to confirm that BLI levels in GFAP-luc animals parallel increases in GFAP expression. Our laboratory has also verified that the amounts of GFAP and luciferase mRNA correspond closely in the brains of these transgenic animals (Gaindh et al., In review). These results correspond with studies using this technique in other neurodegenerative transgenic models including amyotrophic lateral sclerosis, AD and prion disease (Zhu et al., 2004; Luo et al., 2007, 2008; Cordeau et al., 2008; Keller et al., 2009; Tamguney et al., 2009). In the transgenic (SOD1G93A/GFAP-luc) mouse model of amyotrophic lateral sclerosis, BLI revealed multiple phases of increased luminescence that closely correlate with significant increases in GFAP expression (Keller et al., 2009). A rise in luminescence signal measured in prion-inoculated mice with the GFAP-luc transgene corresponded to an increase in GFAP mRNA as well as IHC for GFAP, indicative of reactive astrocytic gliosis (Tamguney et al., 2009). In bigenic mouse models of AD, an age-dependent increase in bioluminescence signals correlated with the astrogliosis accompanying the deposition of amyloid in the brain (Watts et al., 2011). Furthermore, Sydow et al. (2016) showed that gliosis in the transgenic Ala152-thr-tau mouse model of tauopathy was accompanied by increased GFAP-luc luminescence. None of these studies reported that insertion of the GFAP-luc gene interfered with disease progression and our own experience examining P301S mice lacking the GFAP-luc transgene show similar clinical deterioration.

The BLI technique that we use is limited in its ability to distinguish the brain structures in which gliosis occurs because it is a comparatively low resolution technique, and our apparatus could not perform three-dimensional reconstruction. However the regional distribution of astrocytosis revealed by BLI in this study is consistent with earlier studies of P301S mice that reported elevated gliosis in the forebrain (Yoshiyama et al., 2007; Hampton et al., 2010; López-González et al., 2015; Ji et al., 2018), hindbrain and spinal cord (López-González et al., 2015). Similarly our findings that bioluminescence rises between 4 and 7 months of age corresponds to those of Yoshiyama et al. (2007) and Hampton et al. (2010) who found that P301S mice had relatively low levels of GFAP at 2–3 months with sharp increases at 6 months of age, as well as a similar time course of hyperphosphorylated tau accumulation. On the other hand, López-González et al. (2015) concluded that GFAP expression in P301S mice was minimal at 6 months, but higher at 10 months, well after the age when hyperphosphorylated tau became apparent.

Our data show a sharp reduction in brain BLI between 7.5 and 8.5 months in P301S SHAM mice. This may be partially due to a decrease in the size of gliotic brain structures. Yoshiyama et al. (2007) reported that the hippocampal volume of P301S mice shrank by approximately 25% between 6 and 9 months old, accompanied by a small decrease in cortical size. However, we observed a 58% reduction in brain BLI in P301S sham mice between 7.5 and 8.5 months, suggesting that brain atrophy cannot be the sole cause of the decrease. An advantage of the BLI technique is that it allows a simple and detailed longitudinal study of gliosis, in contrast to other studies of P301S mice which were limited in their ability to detect fluctuations in gliosis since they examined only a few time points. However, the data presented by López-González et al. (2015; Figure 5) suggests a rise in gliosis in P301S relative to wild type mice at 3 months old followed by a reduction at 6 months.

BLI techniques have also been used to identify transient rises in gliosis in another transgenic model of neurodegeneration. Watts et al. (2011) examined a mutant amyloid precursor protein-based mouse model of AD with a GFAP-luc transgene [Tg(CRND8-GFAP-luc)]. These animals showed a spike in BLI at 7–8 months. Subsequently, the BLI dropped (while still remaining above baseline) then displayed a second spike at 12 months. In the present study, the brain BLI levels appeared to remain above baseline after the spike at 7.5 months, though the results were not significant at 8.5 and 9 months. It is possible that we would have observed additional BLI spikes if mice had been allowed to survive for a longer period. It should also be noted that Watts et al. (2011) did not observe these transient spikes in a second model of APP pathology [Tg(APP23-GFAP-luc)]. In another study, Luo et al. (2008) described a transient spike in BLI in experimental autoimmune encephalomyelitis in GFAP-luc mice. This model of multiple sclerosis produces a temporary autoimmune reaction with accompanying astrogliosis and is dissimilar to the chronic neurodegenerative model that we use. However, these authors provided a direct demonstration that a transient spike in BLI may be correlated directly to a temporary increase in GFAP IHC.

No single technique can measure all aspects of the progression of a neurodegenerative disease. It is, therefore, not surprising that we observed a disparity between the decrease in brain BLI levels after 7.5 months and the continuous rise in the clinical deficit. The astrocytosis associated with BLI is only one aspect of the P301S model of tauopathy, and BLI should be used in combination with other techniques.

The detailed longitudinal study of BLI also allowed us to correlate the development of gliosis with clinical deficit. In our current model, increased luminescence signals were apparent in the brain as early as 5 months old and matched the onset of clinical deficits in both groups of P301S mice through 7 months of age. Increased luminescence signal also correlated with higher clinical scores in sham-treated vs. JM4-treated mice, suggesting the role of BLI as a valuable tool in following clinical deficit. However, clinical scores continued to rise in mice after 7 months of age whereas bioluminescence stabilized or declined, suggesting that the value of astrogliosis-linked BLI in advanced stages of the disease is limited when other factors become predominant.

Another major strength of our study was our demonstration of the effect of JM4 on disease progression in P301S mice. The results of our study strongly support our hypothesis that JM4 slows the development of deficits in P301S mice through immune modulation. JM4 treated mice showed far slower clinical deterioration than sham-treated mice. This therapeutic effect was mirrored in lower luminescence values in the brain and spinal cord and decreased hippocampal tauopathy. The effect of JM4 cannot be attributed to a general modulation of astrogliosis regardless of disease status since it did not affect BLI levels in 4-month-old P301S mice (2 months after JM4 was initiated, and before the transgene had caused pathological BLI changes in untreated P301S mice). Chronic neuroinflammation is a hallmark of AD, and there is increasing interest in targeting this process for treatment of this disease (McCaulley and Grush, 2015; McGeer et al., 2016; Van Eldick et al., 2016). Furthermore, Yoshiyama et al. (2007) have provided direct evidence that chronic immune suppression reduces tauopathy and hippocampal degeneration in P301S mice. In a previous study on JM4 in experimental autoimmune encephalomyelitis (Yuan et al., 2015), we demonstrated that this treatment blocked dendritic cell proliferation and this was associated with a decrease in Th17 cells, proinflammatory cytokines, and increase in CD4 FoxP3 Treg cells. The current study provides a limited picture of the effects of JM4 treatment on disease development in P301S mice as we did not study effects on memory, motor deficit or longevity. Ongoing studies in our laboratory are examining these factors, as well as whether delayed administration of JM4 can diminish disease signs after they have been established. This study will also examine the effect of JM4 on development of cognitive deficits with early or later JM4 treatment.

The current study can provide only limited information of the mechanism of action of JM4 in the P301S mice; however comparison to prior studies provides some insight. Unpublished results in our laboratory have found that JM4 treatment does not affect total tau levels in P301S mice (Choi et al., in review), therefore reduced tauopathy is not reflective of a general decrease in tau transgene expression. Studies indicating a role of inflammatory microglia in driving tauopathy suggest a possible mechanism. An increase in inflammatory microglia is well established in tauopathy patients (Wes et al., 2016), particularly in brain areas where abnormal tau expression is prominent (Gerhard et al., 2004). In addition, inflammatory microglia proliferate before tauopathy develops in P301S mice (Yoshiyama et al., 2007). Recent studies using knockout mice lacking the microglial CX3Cr1 receptor provide a more direct link between microglia and tauopathy. Knockout of this receptor in mice that overexpress human tau causes increased microglial activation (Bhaskar et al., 2010; Maphis et al., 2015). In addition, adoptive transfer of microglia lacking CX3CR1 induces tauopathy in wild type mice (Maphis et al., 2015). Similarly knockout of the TREM2 receptor in P301S mice caused a reduction of microgliosis, astrogliosis and neurodegeneration (although the reduction of hippocampal tau phosphorylation in this study fell just short of statistical significance; Leyns et al., 2017). Furthermore, eliminating the proinflammatory enzyme 5-lipoxygenase in P301S mice reduces neuroinflammation and tauopathy (Vagnozzi and Pratico, 2018) and its overexpression has the opposite effect (Vagnozzi et al., 2018). The anti-inflammatory environment induced by JM4 seems to reduce microglial activation in the current model and thereby reduces the severity of tauopathy.

In conclusion, our results validate the use of BLI to serially monitor neuropathological changes in tauopathy related models of neurodegeneration. Importantly, they equally validate the beneficial effects of JM4 in countering the inflammatory degenerative condition in the brains of P301S mice and indicate that it may contribute towards the treatment of AD. Together these findings underscore the advantage of BLI as a highly useful biomarker for investigating neurodegenerative diseases.

## Data Availability

All datasets generated for this study are included in the manuscript and/or the Supplementary Files.

## Ethics Statement

Animal use was approved and monitored by the Institutional Animal Care and Use Committee of the Veterans Administration New Jersey Health Care System (VANJHCS), East Orange Campus. This group ensured that the research complied with the ethical guidelines of the United States Public Health Service and the Office of Laboratory Animal Welfare. All animal work was performed within the VANJHCS, East Orange, which is fully accredited by the Association for Assessment and Accreditation of Laboratory Animal Care.

## Author Contributions

WL, AD-M, Y-BC, BB and DG prepared the original draft. PD performed conceptualization and supervision. WL, ER and MM performed the investigation. WL and AD-M performed data analysis. All authors reviewed and edited the manuscript.

## Disclaimer

The contents do not represent the views of the U.S. Department of veterans Affairs for the United States Government.

## Conflict of Interest Statement

PD holds patents on the use of JM4 as an immune/inflammatory modulator in the treatment of brain disorders. The remaining authors declare that the research was conducted in the absence of any commercial or financial relationships that could be construed as a potential conflict of interest.

## Abbreviations

BLI: Bioluminescence imaging
EPO: Erythropoietin
FTD: Frontotemporal dementia
GFAP: glial fibrillary acidic protein
PBS: Phosphate buffered saline
photons/s/cm^2^/sr: Photons per second per square centimeter pre steradian
Tg: transgenic.
